# Porcine Milk-Derived Small Extracellular Vesicles Promote Intestinal Immunoglobulin Production through pIgR

**DOI:** 10.3390/ani11061522

**Published:** 2021-05-24

**Authors:** Bin Zeng, Hailong Wang, Junyi Luo, Meiying Xie, Zhengjiang Zhao, Xingping Chen, Dongyang Wang, Jiajie Sun, Qianyun Xi, Ting Chen, Yongliang Zhang

**Affiliations:** National Engineering Research Center for Breeding Swine Industry, Guangdong Provincial Key Laboratory of Animal Nutrition Control, Guangdong Laboratory for Lingnan Modern Agriculture, College of Animal Science, South China Agricultural University, Guangzhou 510642, China; zengbinyx@163.com (B.Z.); wanghailong03@stu.scau.edu.cn (H.W.); luojunyi@scau.edu.cn (J.L.); xiemeiying2021@163.com (M.X.); orcsblade@163.com (Z.Z.); cxp641972323@163.com (X.C.); wangdy@stu.scau.edu.cn (D.W.); jiajiesun@scau.edu.cn (J.S.); xqy0228@scau.edu.cn (Q.X.)

**Keywords:** extracellular vesicles, milk, exosomes, circRNA, intestinal SIgA

## Abstract

**Simple Summary:**

As the irreplaceable nutrient source for young mammals, milk has a number of biological functions. Milk derived extracellular vesicles are a recently discovered component of milk and have potential regulatory effects on intestinal health and immunity. In this study, in vivo and in vitro experiments were performed to examine the effects of porcine milk small extracellular vesicles (PM-sEVs) on intestinal immunity. As a result, PM-sEVs promoted intestinal secretory immunoglobulin A (SIgA) levels, and increased the expression levels of the polymeric immunoglobulin receptor (pIgR) both in mice and piglet. We identified circ-XPO4 in PM-sEVs as a crucial circRNA, which promotes the expression of pIgR via the suppression of miR-221-5p in the intestinal cell. In conclusion, our research provides a new understanding of the nutritional physiology of porcine milk in intestinal immunity.

**Abstract:**

Secretory immunoglobulin A (SIgA) plays an important role in gut acquired immunity and mucosal homeostasis. Breast milk is the irreplaceable nutritional source for mammals after birth. Current studies have shown the potential functional role of milk-derived small extracellular vesicles (sEVs) and their RNAs cargo in intestinal health and immune regulation. However, there is a lack of studies to demonstrate how milk-derived sEVs affect intestinal immunity in recipient. In this study, through in vivo experiments, we found that porcine milk small extracellular vesicles (PM-sEVs) promoted intestinal SIgA levels, and increased the expression levels of polymeric immunoglobulin receptor (pIgR) both in mice and piglet. We examined the mechanism of how PM-sEVs increased the expression level of pIgR in vitro by using a porcine small intestine epithelial cell line (IPEC-J2). Through bioinformatics analysis, dual-luciferase reporter assays, and overexpression or knockdown of the corresponding non-coding RNAs, we identified circ-XPO4 in PM-sEVs as a crucial circRNA, which leads to the expression of pIgR via the suppression of miR-221-5p in intestinal cells. Importantly, we also observed that oral administration of PM-sEVs increased the level of circ-XPO4 and decreased the level of miR-221-5p in small intestine of piglets, indicating that circRNAs in milk-derived sEVs act as sponge for miRNAs in recipients. This study, for the first time, reveals that PM-sEVs have a capacity to stimulate intestinal SIgA production by delivering circRNAs to receptors and sponging the recipient’s original miRNAs, and also provides valuable data for insight into the role and mechanism of animal milk sEVs in intestinal immunity.

## 1. Introduction

The gut is not only the core organ to absorb nutrients, but also provides a robust mucosal immune barrier and protection for the body [1]. Among various intestinal defense mechanisms, secretory IgA (SIgA) is mainly involved in protecting intestinal epithelium from toxins and pathogens [2,3]. The production and secretion of intestinal SIgA are complex. Intestinal SIgA is generated by the cooperation of two different types of cells in the intestinal mucosa. Firstly, dimeric IgA (dIgA) containing the joining (J) chain is produced by plasma cells. Then, intestinal epithelial cells (IECs) transport dIgA into the lumen. Transcytosis of dIgA is mediated by the polymeric immunoglobulin receptor (pIgR) [4]. Since each molecule of SIgA transported into the lumen needs to consume one pIgR molecule, the expression of pIgR is closely related to the continuous supply of SIgA [5].

In infant and young mammals, intestinal immune defense plays a crucial role in healthy development in the long-term. As the main source of food for humans and other mammals after birth, breast milk contains multiple bioactive substances. A previous study has shown that breast-fed infants had higher fecal SIgA level than formula-fed infants at four weeks of age [6], which suggests that breast milk is involved in improving intestinal mucosal immunity in early infancy. Although immunologically active factors and the oligosaccharides remain the focused fields of breast milk research, recent studies have shown that milk contains abundant small extracellular vesicles (sEVs), which may also contribute to the short- and long-term benefits of intestine [7]. sEVs are a heterogeneous group of secreted nanoparticles, such as “exosomes”, and act as message transmitters in intercellular or inter-organizational communication because they contain a lot of bioactive cargos [8].

Among sEVs cargos, noncoding RNAs (ncRNAs) have received a lot of attention. Exosomes (a type of sEVs) can transport ncRNAs, including microRNAs (miRNAs) [9], long noncoding RNAs (lncRNAs) [10], and circular RNAs (circRNAs) [11], from donor to recipient cells, and perform corresponding biological functions. There is a growing body of evidence that milk sEVs of mammals contain abundant ncRNAs [7]. Due to the protective action of the lipid bilayer of milk sEVs, their ncRNA cargos are stable and resistant to harsh conditions, including gastrointestinal digestive juices, freeze–thaw cycles, and RNase digestion [12,13,14]. Studies have shown that milk sEVs promote the growth of IECs [15] and reduce intestinal injury induced by oxidative stress [16,17] and necrotizing enterocolitis [18]. Milk sEVs-derived RNAs can be absorbed by macrophages and intestinal cells in vitro [19,20], and can enter the circulatory system of milk consumers in vivo [21]. Recent research has shown that bovine milk exosomal miR-200a-3p is involved in alleviating cecal inflammation in mice [22].

As the main source of nutrition for piglets, porcine milk is a key component in the pig breeding industry. Our team has isolated porcine milk sEVs (PM-sEVs), and identified their miRNAs as potential regulatory molecules in mother-to-offspring relation [23]. Our previous studies have shown that PM-sEVs facilitated intestinal tract development in vivo and in vitro [24]. PM-sEVs carrying miR-4334, miR-338, and miR-219 reduce lipopolysaccharide-induced IECs inflammation and apoptosis via the NF-κB and p53 pathway [25]. PM-sEVs carrying miR-365-5p and miR-30c attenuate deoxynivalenol-induced IECs damage by downregulating the expression of protein 53 and Serine proteinase inhibitor type-1 [26]. Furthermore, our recent work has shown that PM-sEVs contain lncRNAs and circRNAs, and many circRNAs were predicted to be involved in regulating intestinal barrier and mucosal immunity by targeting corresponding miRNAs [27]. CircRNAs are classified as endogenous noncoding RNAs, and are characterized by covalently closed loops. Recent studies have shown that sEVs circRNAs regulate gene expression in recipient cells by acting as miRNA sponges [11,28].

Up to now, how milk-derived sEVs influence SIgA remains unexplored. Based on current knowledge, we hypothesized that PM-sEVs and their ncRNA cargos may play a role in SIgA production in the intestine. In this study, in vivo and in vitro experiments were performed to examine the effects of PM-sEVs on intestinal immunity. We demonstrated that PM-sEVs promote intestinal SIgA production. We further identified circ-XPO4 in PM-sEVs as a crucial circRNA that promotes the expression of pIgR in the piglet intestine.

## 2. Materials and Methods

### 2.1. Milk Small Extracellular Vesicles Isolation

Fresh porcine milk was obtained from healthy Landrace sows within 3–5 days after delivery at a pig farm in Guangdong, China. A combination with fractional centrifugation and ultracentrifugation was used to separate PM-sEVs. To remove fat, somatic cells, cell debris, and partial casein, fresh milk samples were centrifuged at 2000× *g* and 15,000× *g* for 30 min at 4 °C, respectively. In the stage of centrifugation at 2000× *g*, somatic cells in the lowest layer are collected for Western Blotting analysis. The supernatant passed through a 0.45 μm filter to get a clear whey fraction. The clear whey fraction was ultra-centrifuged at 120,000× *g* for 90 min at 4 °C, and the pellets were re-suspended in phosphate-buffered saline (PBS) and then passed through a 0.22 μm filter to get milk sEVs.

### 2.2. Transmission Electron Microscopy (TEM), Particle Size and Protein Concentration Analysis

The morphology of PM-sEVs was observed by TEM. Briefly, the PM-sEVs sample was set at the copper grid coated with formvar for 2 min, washed with ultrapure water, negatively stained with 1% uranyl acetate, observed and photographed with JEOL, JEM2000EX transmission electron microscopy (JEOL, Tokyo, Japan). The size of PM-sEVs was analyzed via the Zetasizer Nano ZS 90 system (Malvern, UK). Protein concentration of Milk sEVs was measured by BCA Protein Assay Kit.

### 2.3. Animal Experiments and Sample Collection

For mouse experiments, sixteen weaned C57BL/6 male mice (three weeks of age) were purchased from Guangdong Animal Experimental Center. Randomly, the mice were grouped into a control group and the PM-sEV group, equal in number. All mice were fed with the custom AIN-93G diet. The diet contains no milk ingredients, and the composition was showed in Table S1. Each mouse was kept separately in a cage, housed in a room with a temperature of 25 ± 2 °C, a photoperiod of 12/12 h (day/night), and a relative humidity of 60 ± 10%. All mice had free access to water and food. In the following 21 days, PM-sEVs (containing 0.4 mg protein, purified from 0.2 mL porcine milk) were administered orally by gavage to the PM-sEVs group daily, while the control group mice were administered SPI solution (ingredients in the diet) of the same volume and protein amount each day. On day 22, the mice were sacrificed and their intestinal tissue and luminal contents of jejunum were taken. These samples were frozen in liquid nitrogen and stored at −80 °C for use in enzyme linked immunosorbent assay (ELISA), quantitative real-time PCR (qRT-PCR), and Western Blotting analyses. In addition, a length of intestine tissue was fixed in 4% paraformaldehyde for immunofluorescence test.

For piglet experiments, twelve male Landrace piglets just after birth without sucking any colostrum or vaccination were purchased from Shuitai pig farm (Yunfu, Guangdong). All piglets with a birth weight of 1.3–1.5 kg were randomly divided into control group (fed milk powder, Pigipro 1 START-Piglet Milk, Schils Group; the nutritional composition of the milk powder is shown in Table S2), and the PM-sEVs group (fed milk powder and PM-sEVs, PM-sEVs are mixed into the prepared liquid milk). All of the piglets were housed in a room maintained under a temperature of 38 °C, and a relative humidity of 60 ± 10%. The milk powder was fed every three hours. The PM-sEVs group was supplied with PM-sEVs (containing 500 mg protein, purified from 250 mL porcine milk) per day. After seven days, the piglets were sacrificed and their intestinal tissue and luminal contents of jejunum were taken. These samples were frozen in liquid nitrogen and stored at −80 °C for use in ELISA, qRT-PCR, and Western Blotting analysis.

### 2.4. Cell Lines and Cell Culture

IPEC-J2 cells in use were a kind gift from Professor Yulong Yin at the Institute of Subtropical Agriculture, Chinese Academy of Sciences. IPEC-J2 cells and HEK-293T cells (from ATCC) were cultured in DMEM/F-12 and DMEM/high glucose (Invitrogen, Life Technologies, Carlsbad, CA, USA), respectively. Two cell lines were supplemented with 10% fetal bovine serum (GIBCO) in cell culture, and maintained at 37 °C in a humidified atmosphere that contained 5% CO_2_.

### 2.5. Milk sEVs Treatment in IPEC-J2 Cells

IPEC-J2 cells were seeded in 12-well plates with 1 × 10^5^ cells per well. After the cells reached 70–80% confluency (about 16 h after seeding), they were treated with a control solution (PBS) or 100 μg/mL of PM-sEVs. The cells were harvested at 24 h, 48 h after treatment for qRT-PCR and Western Blotting analysis, respectively.

### 2.6. Bioinformatics Analysis and Dual-Luciferase Reporter Assay

Target gene prediction was performed by the software of miRanda [29] and RNAhybrid [30]. The sequences of circ-XPO4 (containing the miR-221-5p binding regions, or parts of the mutated and deleted) and 3′UTR of pIgR (containing the miR-221-5p binding regions, or parts of the mutated and deleted) were synthesized and inserted into a pmirGLO vector to construct the pmirGLO-circ-XPO4 and pmirGLO-pIgR vectors, respectively. HEK-293T cells (3 × 10^4^ cells per well) were seeded in 96-well cell culture plates. After the cells reached 70–80% confluency, cells were transfected with recombinant pmirGLO-pIgR vector or pmirGLO-circ-XPO4 vector (100 ng/well) mixed with their corresponding miR-221-5p mimics or NC (3 pmol/well) using the Lipofectamine 3000 reagent (Invitrogen, Carlsbad, CA, USA) according to the manufacturer’s instructions for 24 h. Then, luciferase activity was detected by a dual luciferase reporter assay system (Promega, Madison, WI, USA).

### 2.7. Cell Transfection

MiR-221-5p mimics/inhibitors and small interfering RNA (siRNA) overlapping junction sites of circ-XPO4 (si.circ-XPO4, 5′-CCGATGTACCACTTCCTCCTT-3′) were synthesized (GenePharma, Shanghai). For circ-XPO4 overexpression plasmid construction, the linear sequence of circ-XPO4 was synthesized and cloned into pCD2.1-ciR (Geneseed Biotech, Guangzhou, China) using the KpnI and BamHI restriction sites. IPEC-J2 cells were seeded in 12-well plates with 1 × 10^5^ cells per well. After the cells reached 70–80% confluency, they were transfected with miR-221-5p NC/mimics/inhibitors (30 pmol/well) and si.NC/si.circ-XPO4 (40 pmol/well, under the condition of 100 μg/μL PM-sEVs treatment) or OE.NC/OE.circ-XPO4 (1μg/well) using the Lipofectamine 3000 reagent according to the manufacturer’s instructions. The cells were harvested at 24 h, 48 h after transfection for qRT-PCR and Western Blotting analysis, respectively.

### 2.8. Validation of PM-sEVs circRNAs

PM-sEVs total RNAs were extracted by Trizol reagent. RNA was reverse transcribed using a PrimeScript™ RT Reagent Kit (Takara, Dalian, China). PCR (a pair of convergent and divergent primers) and Sanger sequencing were used to verify the head-to-tail splicing junction of circRNA. We also performed RNase R treatment. 2 μg of PM-sEVs total RNA was incubated with 3 U/μg RNase R (Geneseed Biotech, Guangzhou, China) or RNase-free water (as a control) at 37 °C for 10 min. The expression levels of circRNA and linear mRNA (β-actin) were detected by PCR and agarose gel electrophoresis. Table S3 shows the primer sequences used in PCR analysis.

### 2.9. Enzyme-Linked Immunosorbent Assay

Intestinal lumen contents of mice and piglets were diluted with PBS containing 1 mM phenylmethylsulfonyl fluoride (PMSF) at a ratio of 1:10 (*w*/*v*). These samples were homogenized, and centrifuged at 12,000× *g* for 5 min at 4 °C. The supernatant was obtained to measure the levels of SIgA using corresponding ELISA kit.

### 2.10. Immunofluorescence

The intestinal tissue fixed with paraformaldehyde was embedded in paraffin and made into 5-μm sections. The sections were incubated with rabbit anti-IgA (bs-0774R, Bioss, Beijing, China) for 12 h at 4 °C. After washing with PBS for three times, the sections were incubated with FITC-conjugated secondary antibody (bs-0295G, Bioss, Beijing, China) for 1 h. The cell nuclei were stained with DAPI (H-1200, Vector Laboratories). Images were observed and photographed with Nikon Eclipse Ti-s microscope (Tokyo, Japan).

### 2.11. Gene Expression Analysis Using qRT-PCR

Total RNA was extracted from PM-sEVs, intestinal tissues, and IPEC-J2 cells using the TRIzol reagent. The cDNA (used to analyze mRNAs and circRNAs) was synthesized using a PrimeScript™ RT Reagent Kit (Takara, Dalian, China). The cDNA (used to analyze miRNAs) was synthesized using a Mir-X miRNA First-Strand Synthesis Kit (Takara, Dalian, China). All assays were performed according to the manufacturer’s protocols. The qRT-PCR was performed using the SYBR Green Mix reagents (Promega, Madison, WI, USA) and CFX96 Touch™ detection instrument (BIO-RAD, Hercules, CA, USA). The expression of circRNAs and mRNAs was normalized to *Actb* (β-actin), and the expression levels of miRNAs were normalized to U6, using the 2^−ΔΔCt^ method. Primer information is given in Tables S3 and Table S4.

### 2.12. Western Blotting Analysis

The protein samples were extracted from milk sEVs, milk somatic cells, intestinal tissues, and IPEC-J2 cells using radio immunoprecipitation assay lysis buffer containing PMSF protease inhibitor. The protein samples were homogenized, and centrifuged (12,000× *g*, 5 min, 4 °C). The supernatant was obtained to further analysis. Protein concentration of the samples was measured by BCA Protein Assay Kit. Protein samples were mixed with sodium dodecyl sulfate loading buffer and denatured in a boiling water bath. The protein samples were added to 10% SDS-PAGE gel for separation and then transferred to polyvinylidene fluoride membranes. The membranes were blocked with 5% skim milk for 2 h and incubated with specific antibodies for 12 h at 4 °C. The following antibodies were used: anti-CD63, anti-TSG101, anti-Alix, and anti-Calnexin (Sangon Biotech, Shanghai, China), anti-pIgR-mice (AF2800-SP, R&D Systems, Minneapolis, MN, USA), anti-pIgR-pig (bs-6061R, Bioss, Beijing, China), and anti-β-actin (AP0060, Bioworld, Technology Inc., Bloomington, MN, USA). Then, the membranes were incubated with HRP-conjugated corresponding secondary antibodies for 1 h. Images of protein bands were visualized using FluorChem M (ProteinSimple, San Jose, CA, USA). Gray value of protein bands were analyzed using Image J software.

### 2.13. Statistical Analysis

All data were analyzed using one-way ANOVA (multiple comparisons) and *t*-test (pairwise comparisons) in SPSS 17.0 (SPSS Inc., Chicago, IL, USA). All data are expressed as means ± SEM. *p* < 0.05 was considered statistically significant.

## 3. Results

### 3.1. Identification of PM-sEVs

**Figure 1:**
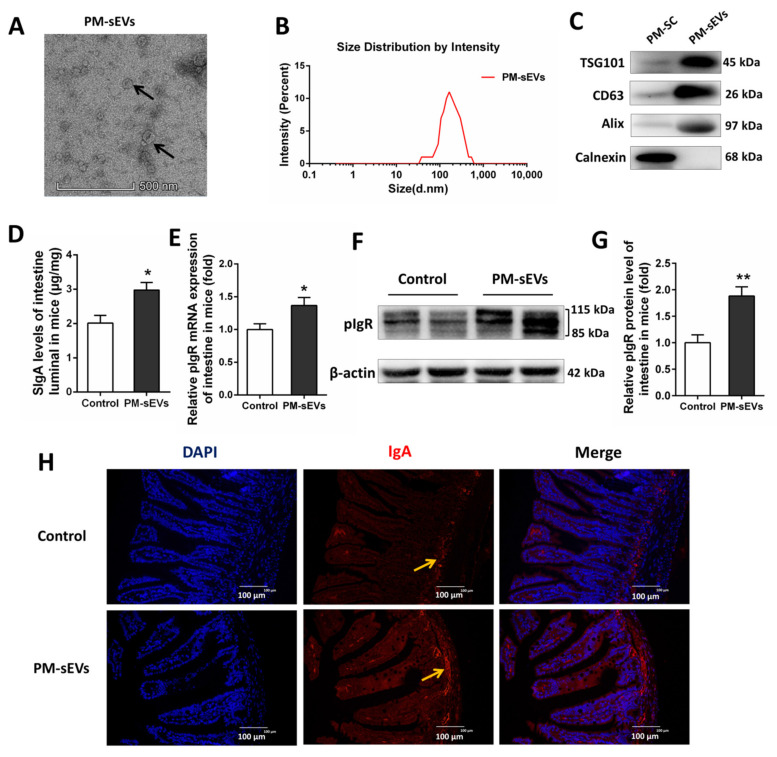
Identification of PM-sEVs and effect of PM-sEVs on intestinal SIgA levels in mice. (**A**) Morphology of PM-sEVs visualized under TEM. Scale bar: 500 nm. The arrows indicate “PM-sEVs”. (**B**) Size distribution analysis of PM-sEVs. (**C**) Western Blotting analysis of porcine milk somatic cell (PM-SC) and PM-sEVs. Milk SC and PM-sEVs were blotted for commonly used sEVs markers TSG101, CD63, and Alix and the endoplasmic reticulum marker Calnexin. (**D**) ELISA analysis of SIgA levels of intestinal luminal contents in mice (*n* = 8). (**E**) qRT-PCR analysis of pIgR mRNA expression levels of intestinal tissue in mice (*n* = 8). (**F**,**G**) Western Blotting analysis of pIgR protein level of intestinal tissue in mice (*n* = 4). (**H**) Intestine of mice was sectioned for immunofluorescence staining (Scale bar = 100 μm) to analysis the level of IgA (*n* = 4). All data are presented as means ± SEM. * *p* < 0.05; ** *p* < 0.01.

We identified PM-sEVs with reference to MISEV2018 [31]. PM-sEVs exhibited a spherical microvesicle structure observed under TEM (Figure 1A). The results of particle size analysis showed that the average diameters of the PM-sEVs were about 146.9 nm (Figure 1B). The exosomal positive markers TSG101, CD63, and Alix were highly expressed on PM-sEVs rather than porcine milk somatic cell (PM-SC), while Calnexin (resident of the endoplasmic reticulum) only expressed on PM-SC (Figure 1C). Overall, these results confirm the success of sEVs isolated from porcine milk.

### 3.2. PM-sEVs Promote Intestinal Secretory IgA (SIgA) Levels in Mice and Piglet

**Figure 2:**
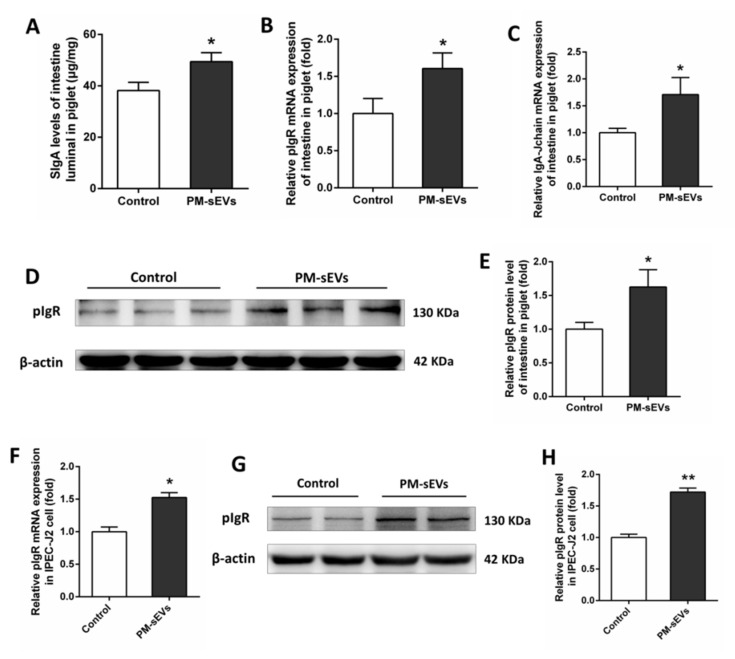
PM-sEVs promote intestinal SIgA levels in piglets and influence relevant gene expression in piglet intestine and IPEC-J2 cells. (**A**) ELISA analysis of SIgA levels of intestinal luminal contents in piglets (*n* = 6). (**B**,**C**) qRT-PCR analysis of IgA-Jchain, pIgR mRNA expression levels of intestinal tissue in piglets (*n* = 6). (**D**,**E**) Western Blotting analysis of pIgR protein level of intestinal tissue in piglets (*n* = 3). (**F**) qRT-PCR analysis of pIgR mRNA expression levels in IPEC-J2 cell (*n* = 6). (**G**,**H**) Western Blotting analysis of pIgR protein levels in IPEC-J2 cell (*n* = 4). All data are presented as means ± SEM. * *p* < 0.05; ** *p* < 0.01.

SIgA is a key molecule in the regulation of intestinal mucosal immune. We analyzed SIgA levels in the intestine of mice via ELISA. Compared with control mice, intestinal SIgA level in PM-sEVs mice was significantly increased (*p* < 0.05, Figure 1D). Immunofluorescence analysis also showed that IgA levels in the intestinal tract in PM-sEV mice were more abundant than those in the control mice (Figure 1H). As a key molecule in the process of SIgA transport, pIgR level was also been examined. qRT-PCR results indicated that PM-sEVs increased the mRNA expression levels of pIgR in intestine of mice (*p* < 0.05, Figure 1E). Western Blotting analysis further confirmed this result (Figure 1F–G). In addition, PM-sEVs were found to have similar effects in piglets. The levels of intestinal SIgA in piglets were increased by supplementing PM-sEVs to the milk powder formula solution (*p* < 0.05, Figure 2A). The mRNA expression levels of pIgR and IgA-Jchain in the piglets’ intestine were increased in the PM-sEVs group (*p* < 0.05, Figure 2B,C). Western Blotting analysis showed that the level of pIgR protein in intestinal tissues of piglet of the PM-sEVs group was higher than that in the control group (*p* < 0.05, Figure 2D,E). All these results indicated that PM-sEVs were able to promote intestinal SIgA level and pIgR expression both in mice and in piglet.

### 3.3. PM-sEVs Promote pIgR Levels in IPEC-J2 Cells

PIgR is produced by IECs. To verify whether PM-sEVs influence pIgR levels in intestinal cells, we determined pIgR expression level of IPEC-J2 cells treated with PM-sEVs in vitro. The results revealed that pIgR mRNA and protein expression levels of IPEC-J2 cells were increased in the group treated with 100 μg/mL PM-sEVs (*p* < 0.05, Figure 2F–H), compared with the control group (treated with PBS). These data suggested that PM-sEVs were able to promote pIgR level in IPEC-J2 cells, which was fully consistent with the in vivo results.

### 3.4. PM-sEVs Influence Noncoding RNA Expression Levels in IPEC-J2 Cells and Piglet Intestine

**Figure 3:**
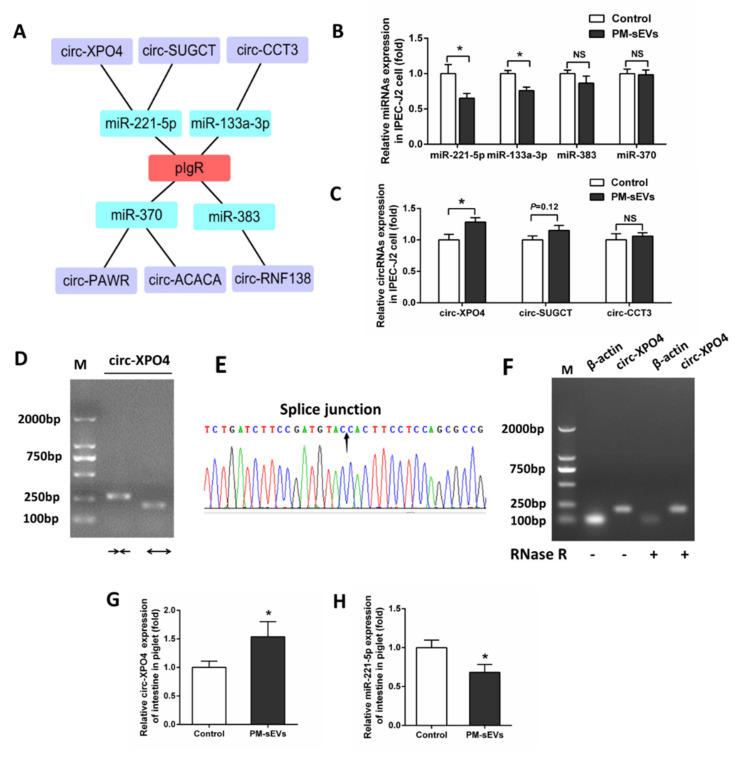
Effects of PM-sEVs on noncoding RNA expression in IPEC-J2 cell and piglet intestine. (**A**) The potential interaction among pIgR, miRNAs, and PM-sEVs circRNAs. (**B**) qRT-PCR analysis of miR-221-5p, miR-133a-3p, miR-383, and miR-370 expression levels in IPEC-J2 cell (*n* = 6). (**C**) qRT-PCR analysis of circ-XPO4, circ-SUGCT, and circ-CTT3 expression levels in IPEC-J2 cell (*n* = 6). (**D**) PCR and agarose gel electrophoresis identify PCR product size of circ-XPO4, “→←” represent convergent primers, “←→” represent divergent primers. (**E**) Sanger sequencing confirm the splice junctions of circ-XPO4, the arrow points to the splice junctions. (**F**) PCR and agarose gel electrophoresis analysis the abundance of circ-XPO4 and linear mRNA (β-actin) treated with RNase R. (**G**,**H**) qRT-PCR analysis of circ-XPO4 and miR-221-5p expression levels in intestinal tissue of piglet (*n* = 6). NS, represent non-significant (*p* > 0.05); * *p* < 0.05. All data are presented as means ± SEM.

As mentioned above, our recent work has shown that many circRNAs in PM-sEVs were predicted to participate in intestinal barrier and mucosal immunity by targeting corresponding miRNAs [27]. By bioinformatics analysis, we found that there were 6 circRNAs in PM-sEVs that could regulate pIgR by binding 4 miRNAs (Figure 3A). qRT-PCR analysis showed that the expression levels of miR-221-5p and miR-133a-3p were decreased after treatment with PM-sEVs (*p* < 0.05, Figure 3B), while the expression levels of miR-383 and miR-370 were not significantly different (Figure 3B). We also analyzed the expression levels of the circRNAs that were predicted to bind the mentioned miRNAs. The results showed that PM-sEVs increased circ-XPO4 expression level, compared with control (*p* < 0.05, Figure 3C). The expression levels of circ-SUGCT and circ-CCT3 were not significantly different between control and PM-sEVs groups (Figure 3C). To certify the circular structure of circ-XPO4, a pair of divergent and convergent primers was used for PCR amplification (Figure 3D). We then verified the junction sequences of circ-XPO4 using Sanger sequencing (Figure 3E). After RNase R treatment of circ-XPO4 and β-actin mRNA, PCR and agarose gel electrophoresis analysis were carried out, and the result showed that the expression levels of circ-XPO4 were not changed between mock and RNase R treatment groups. However, the expression level of β-actin was different (Figure 3F). We also investigated the expression levels of circ-XPO4 and miR-221-5p in piglet intestine using qRT-PCR. Interestingly, the level of circ-XPO4 was increased in PM-sEVs group, while the miR-221-5p level was downregulated (Figure 3G,H). The above data indicated that PM-sEVs promoted pIgR levels in intestine, which may associate with the changed levels of miR-221-5p and circ-XPO4.

### 3.5. Validation of pIgR as a Direct Target of miR-221-5p in IPEC-J2 Cells

**Figure 4:**
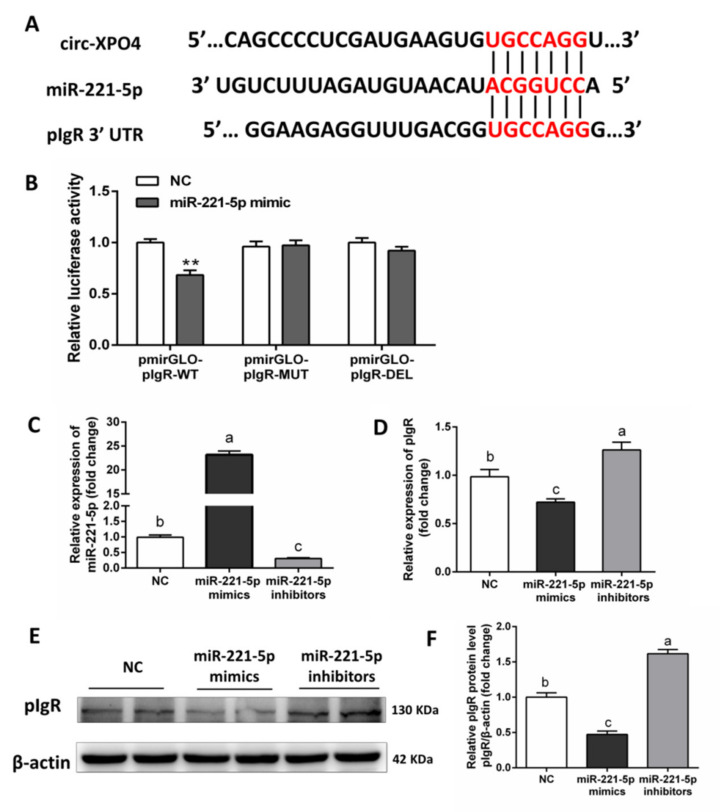
Validation of pIgR as a direct target of miR-221-5P in IPEC-J2 cells. (**A**) Bioinformatics analyses predicted binding Scheme 221. p and pIgR, miR-221-5p and circ-XPO4. (**B**) Dual luciferase reporter system detects the targeting relationship between miR-221-5p and pIgR (*n* = 8). (**C**,**D**) qRT-PCR analysis of miR-221-5p and pIgR expression levels in IPEC-J2 cell treated with NC, miR-221-5p mimics or miR-221-5p inhibitors (*n* = 6). (**E**,**F**) Western Blotting analysis of pIgR protein levels in IPEC-J2 cell treated with NC, miR-221-5p mimics or miR-221-5p inhibitors (*n* = 4). All data are presented as means ± SEM. ** *p* < 0.01. Different superscripts “a”/“b”/“c” represent significant differences between groups (*p* < 0.05).

Bioinformatic analysis showed that the 3′-UTR of pig pIgR harbors the predicted binding site of miR-221-5p (Figure 4A). A dual-luciferase reporter system was used to verify the predicted interactions between miR-221-5p and pIgR. Luciferase reporter plasmids containing the wild type (WT), mutated (MUT) or deleted (DEL) sequences of the miR-221-5p binding site was constructed. HEK-293T cells were co-transfected with the reporter plasmids and synthetic miR-221-5p mimics or NC. The results revealed that miR-221-5p mimics significantly suppressed luciferase activity in pmirGLO-pIgR-WT group, while in the pmirGLO-pIgR-MUT and pmirGLO-pIgR-DEL groups, this inhibition was almost abolished (Figure 4B). To determine whether miR-221-5p inhibit pIgR expression in IECs of piglet, IPEC-J2 cells were transfected with synthetic miR-221-5p mimics, which led to a remarkable increase in miR-221-5p in cells (Figure 4C). miR-221-5p mimics reduced pIgR mRNA abundance (Figure 4D) and protein levels (Figure 4E,F). miRNA inhibitors were used to suppress miR-221-5p expression in IPEC-J2 cells, which trigger the increase of the mRNA and protein expression of pIgR (Figure 4D,F). These results demonstrated that miR-221-5p directly suppressed pIgR in IPEC-J2 cells.

### 3.6. Circ-XPO4 Regulates pIgR Expression via miR-221-5p in IPEC-J2 Cells

**Figure 5:**
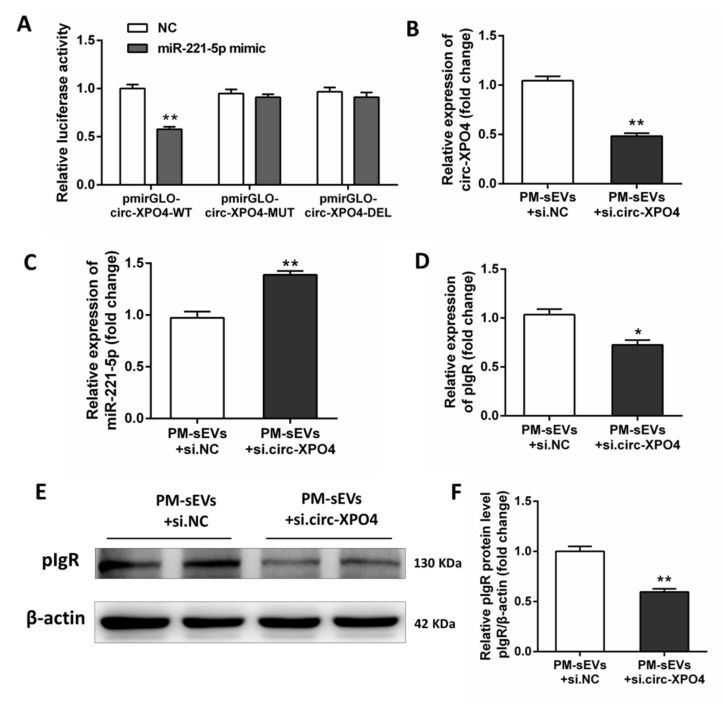
PM-sEVs circ-XPO4 regulates the miR-221-5p/pIgR pathway in IPEC-J2 cells. (**A**) Dual luciferase reporter system detect the targeting relationship between miR-221-5p and circ-XPO4 (*n* = 8). (**B**–**D**) qRT-PCR analysis of circ-XPO4, miR-221-5p, and pIgR expression levels in IPEC-J2 cell treated with PM-sEVs + si.NC or PM-sEVs + si.circ-XPO4 (*n* = 6). (**E**,**F**) Western Blotting analysis of pIgR protein levels in IPEC-J2 cell treated with PM-sEVs + si.NC or PM-sEVs + si.circ-XPO4 (*n* = 4). * *p* < 0.05; ** *p* < 0.01.

**Figure 6:**
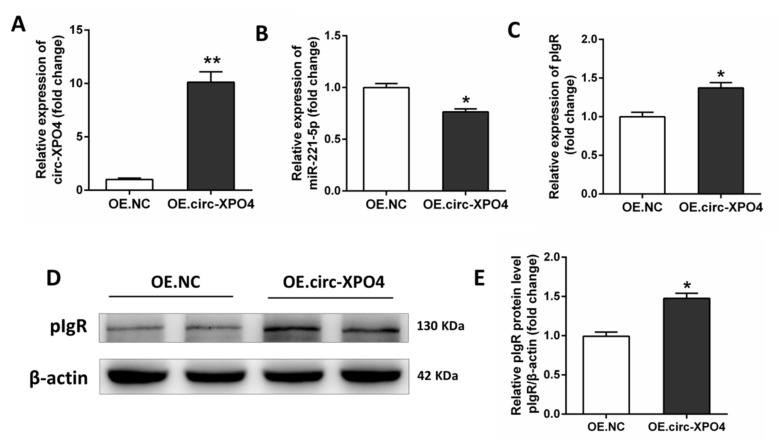
PM-sEVs circ-XPO4 regulates the miR-221-5p/pIgR pathway in IPEC-J2 cells. (**A**–**C**) qRT-PCR analysis of circ-XPO4, miR-221-5p, and pIgR expression levels in IPEC-J2 cell treated with OE.NC or OE.circ-XPO4 (*n* = 6). (**D**,**E**) Western Blotting analysis of pIgR protein levels in IPEC-J2 cell treated with OE.NC or OE.circ-XPO4 (*n* = 4). * *p* < 0.05; ** *p* < 0.01.

The sequences of the binding region between circ-XPO4 and miR-221-5p are shown in Figure 4A, suggesting that circ-XPO4 has a stable interaction with miR-221-5p. We used a dual-luciferase reporter system assay to further demonstrate the direct interactions between circ-XPO4 and miR-221-5p. The results showed that circ-XPO4 directly targeted miR-221-5p (Figure 5A). Subsequently, under PM-sEVs treatment, IPEC-J2 cells were transfected with synthetic siRNA to inhibit circ-XPO4 expression. The expression of miR-221-5p was enhanced when circ-XPO4 was knocked down by siRNA (Figure 5B,C). We transfected overexpressed circ-XPO4 plasmids into IPEC-J2 cells, which leads to significantly increased circ-XPO4 but decreased miR-221-5p level (Figure 6A,B). We used qRT-PCR and Western Blotting to evaluate the effects of circ-XPO4 on pIgR. The mRNA and protein expression of pIgR were significantly inhibited by circ-XPO4 siRNA but were enhanced by overexpressed circ-XPO4 (Figure 5D–F, Figure 6C–E). These results demonstrated that circ-XPO4 acts as a sponge of miR-221-5p to regulate pIgR expression in IPEC-J2 cells.

## 4. Discussions

Unlike cell-derived sEVs, the origin of sEVs in milk remains to be explored. In the current study, somatic cell from milk was used as a control for the first time to identify our isolated milk sEVs. The protein expression of TSG101, CD63, Alix, and Calnexin was different between milk sEVs and somatic cell. In previous study, human milk exosomes (EVs) were also characterized positive for CD9, CD63 and Alix, and negative for Calnexin [32]. These results further enriched the protein validation of milk sEVs. Many studies have confirmed that milk sEVs have a variety of biological functions, including intestinal health and immune regulations. Milk sEVs promote the growth of IECs [15] and reduce intestinal injury induced by oxidative stress [16,17], intestinal inflammation [33], and necrotizing enterocolitis [18,34]. A recent publication showed that camel milk-derived sEVs ameliorate cyclophosphamide-induced immunotoxicity in rats [35]. However, the components of milk sEVs are complex and many are biologically active, and the functions of these components after delivery to the recipient are far from well understood. The abundant non-coding RNAs in milk sEVs may be the key substances to play corresponding biological roles [7]. Here, we proved that administration of PM-sEVs can improve the intestinal SIgA levels in piglets. PM-sEVs circ-XPO4 participated in the process as a crucial circRNA, which leads to the expression of pIgR in intestine.

In our study, we found that PM-sEVs can promote intestinal SIgA secretion in mice. Similar results were revealed in a previous study [36], in which female C57BL/6 mice at 3 weeks of age received bovine milk-derived extracellular vesicles. In that study, however, the mRNA expression level of pIgR in the intestine was not significantly different after administration of bovine milk sEVs. They believed that the rise of intestinal SIgA levels might be attributed to the changes of the relative composition of gut microbiome and SCFAs. As piglets are the direct recipients of porcine milk, we also examined the effect of PM-sEVs on intestinal immunity in piglets, who did not receive any breast milk. The results showed that PM-sEVs increased intestinal SIgA secretion and pIgR expression level both in mice and piglets. As a key molecule in intestinal SIgA transport, pIgR is synthesized by intestinal epithelial cells [4]. IPEC-J2 cell line is derived from the natural piglet small intestine and is very similar to the physiological function of piglet intestinal epithelium [25]. Therefore, IPEC-J2 cell was used in this study. According to the previous experiment of our team [25,26], 100 μg/mL of PM-sEVs was selected as the treatment dose. It is evident that the level of pIgR has increased in IPEC-J2 cells, after treating with PM-sEVs. Combined with the above results both in vivo and in vitro, pIgR is one of the key targets of PM-sEVs to improve intestinal immunity.

Noncoding RNAs are abundant in sEVs and participate in many biological processes. sEVs lncRNAs and circRNAs act as miRNA sponges, leading to suppression of miRNA function in recipient cell [10,28]. Furthermore, our recent work has shown that PM-sEV circRNAs may participate in intestinal barrier and mucosal immunity by targeting corresponding miRNAs [27]. We analyzed PM-sEVs circRNAs by bioinformatics analyses and found that six circRNAs could have potential regulations in the pIgR through four miRNAs. We also verified that PM-sEVs increased circ-XPO4 levels, while decreased miR-221-5p level both in IPEC-J2 cell and piglet intestine, as shown by qRT-PCR. Similarly, it has been experimentally demonstrated that milk sEVs ncRNAs could be absorbed by macrophages [19] and intestinal cells [20] in vitro. We verified that miR-221-5p targets pIgR and circ-XPO4 by a dual-luciferase reporter system. More interestingly, Solexa sequencing showed a total of 491 miRNAs in PM-sEVs. The abundance of miRNA-221-5p in PM-sEVs was extremely low compared to the high abundance of other miRNAs (let-7a and miR-30a) [23]. In this study, we confirmed this result by qRT-PCR (Figure S1). In addition, Western Blotting analysis confirmed that the expression level of pIgR protein in PM-sEVs was extremely low (Figure S2).

SIgA is the most effective component of intestinal productive mucosal immunity. The production of intestinal SIgA is complex and regulated by a lot of factors [2]. In this study, we focused on the effects of PM-sEVs on the pIgR expression. The in vitro experiments showed that overexpressed miR-221-5p substantially inhibited pIgR, while overexpressed circ-XPO4 in IPEC-J2 observably downregulated miR-221-5p, leading to the activation of pIgR. To our knowledge, this is the first report to demonstrate that a circRNA, which is present in PM-sEVs, can act as a miRNA sponge by adsorptive action in recipients, to promote expression of an intestinal SIgA-associated gene.

Milk sEVs are a component of milk comprising a complex mixture, i.e., proteins, lipids, DNA, and RNAs, all of which might have bioactivity [37]. We are still largely ignorant as to whether other components of milk sEVs are involved in regulating intestinal SIgA secretion through different pathways. For instance, gut microbiota play an important role in intestinal SIgA [38]. Although studies have shown that dietary milk sEVs lead to changes in gut microbiota in animal [36,39], none are aimed at clarifying which components in milk sEVs are responsible for this effect. Current literature has reported that miRNAs in milk sEVs have great potential to regulate gut microbiota [40]. We also propose that milk sEVs should be further studied with proteomic analysis in intestinal barrier and mucosal immunity, since milk sEVs can provide immunological (proteomics) perspectives [41,42]. There was still limitation in this study. The expression of pIgR could be determined in vitro when treated with PM-sEVs or associated ncRNA, but there is a lack of SIgA verification in vitro because the secretion of intestinal SIgA requires the participation of different cells. Due to technical and methodological limitations, PM-sEVs with specific ncRNA depletion was not obtained. Therefore, more seminal studies are needed to fully understand the changes in intestinal barrier resulted from the components in milk sEVs.

## 5. Conclusions

In conclusion, the present study demonstrated that PM-sEVs promote intestinal SIgA production both in mice and piglet. PM-sEVs circ-XPO4 plays an important role in IPEC-J2 cell or recipients through the absorption of miR-221-5p and activation of the pIgR. These findings provide a new understanding to the nutritional physiology of porcine milk sEVs by delivering special ncRNA cargos in intestinal immunity, and push the research of the biological functions of non-coding RNAs in animal milk sEVs.

## Data Availability

Data is contained within the article.

## Supplementary Materials

The following are available online at https://www.mdpi.com/article/10.3390/ani11061522/s1, Table S1: Composition of AIN-93G-based soy protein isolate (SPI) diets, Table S2: The nutritional composition of milk powder, Table S3: PCR primers for piglet, IPEC-J2 cell, and PM-sEVs, Table S4: PCR primers for mice, Figure S1: qRT-PCR analysis of let-7a, miR-30a, and miR-221-5p expression levels in PM-sEVs, Figure S2: Western blotting analysis of pIgR protein levels in IPEC-J2 cell and PM-sEVs.

## Abbreviations

SIgA	Secretory immunoglobulin A
PM-sEVs	Porcine milk small extracellular vesicles
PIgR	Polymeric immunoglobulin receptor
IEC	Intestinal epithelial cell
SC	Secretory component
PCR	Polymerase Chain Reaction
ELISA	Enzyme-linked immunosorbent assay
