# Supplementary material for: Porcine Milk-Derived Small Extracellular Vesicles Promote Intestinal Immunoglobulin Production through pIgR
Source: Animals (Basel). 2021 May 24;11(6):1522. doi: 10.3390/ani11061522 (PMC8225040; doi:10.3390/ani11061522)
Supplement: Supplementary file 1 [file animals-11-01522-s001.zip › animals-1194740-supplementary.pdf]

animals

MDPI

Table S1. Composition of AIN-93G-based soy protein isolate (SPI) diets.

Ingredient	Content (g/kg)
Cornstarch	397.468
Soy protein isolate*(≥ 85% protein)	200.000
Dextrinized cornstarch	132.000
Sucrose	100.000
Soybean oil (no additives)	70.000
Fiber	50.000
Mineral mix (AIN-93G-MX)	35.000
Vitamin mix (AIN-93-VX)	10.000
L-Cystine	3.000
Choline bitartrate (41.1% choline)	2.500
Tert-butylhydroquinone	0.014

* The essential amino acids were added to match the casein levels.

Table S2. The nutritional composition of milk powder.

Nutritional composition	Content (%)
Protein	24.00
Crude Fat	18.00
Ash	6.50
Moisture	4.00
Crude fibre	0.10

Per kg contain Vitamins: vitamin A (50000 IU/kg), vitamine B1 (500 mg/kg), vitamine B2 (100 mg /kg), vitamine B3 (150 mg /kg), vitamin B6 (300 mg /kg), vitamine B12 (100 mg /kg), vitamin C (100 mg /kg), vitamine D3 (15000 IU /kg), vitamin E (300 mg /kg), vitamin K (33 mg /kg).

Table S3. PCR primers for piglet, IPEC-J2 cell, and PM-sEVs.

Gene	Forward (5'-3')	Reverse (5'-3')
β-actin	CCAGCACCATGAAGATCAAGATC	ACATCTGCTGGAAGGTGGACA
pIgR	GGAGATGGCAAATGTGGC	TTTTCCTTGGGAGTGAGCA
circ-XPO4 (convergent)	TCACAAAGGACGAGAACAGC	CATCGGAAGATCAGAGAAGATT
circ-XPO4 (divergent)	GAAGTGTGCCAGGTAGTCAA	GCTGTTCTCGTCCTTTGTGA
circ-SUGCT	TGCTCCAAGGTCTCCTAAAT	ACGTCCCTGGCAAGCTGTCT
circ-CTT3	TGTAAAGACCCCAAAGCCTG	CATCTCCCCCGCAAGAATAA
IgA-Jchain	AAGAAAGCAGAAGCACCGT	CTCAACAAAAATGACCAGGAC
miR-133a-3p	TTGGTCCCCTTCAACCAGCTG	Universal primers (mRQ 3′ Primer)
miR-221-5p	ACCTGGCATACAATGTAGATTTCTGT	Universal primers (mRQ 3′ Primer)
miR-370	GCCTGCTGGGGTGGAACCTGGT	Universal primers (mRQ 3′ Primer)
miR-383	CCACAGCACTGCCTGGTCAGA	Universal primers (mRQ 3′Primer)
Let-7a	GGGTGAGGTAGTAGGTTGT	Universal primers (mRQ 3′ Primer)
miR-30a	GGGCTTTCAGTCGGATGTT	Universal primers (mRQ 3′ Primer)

Table S4. PCR primers for mice.

Gene	Forward (5'-3')	Reverse (5'-3')
β-actin	TGCTGTCCCTGTATGCCTCT	CTTTGATGTCACGCACGATTT
pIgR	AGTAACCGAGGCCTGTCCTT	GTCACTCGGCAACTCAGGA

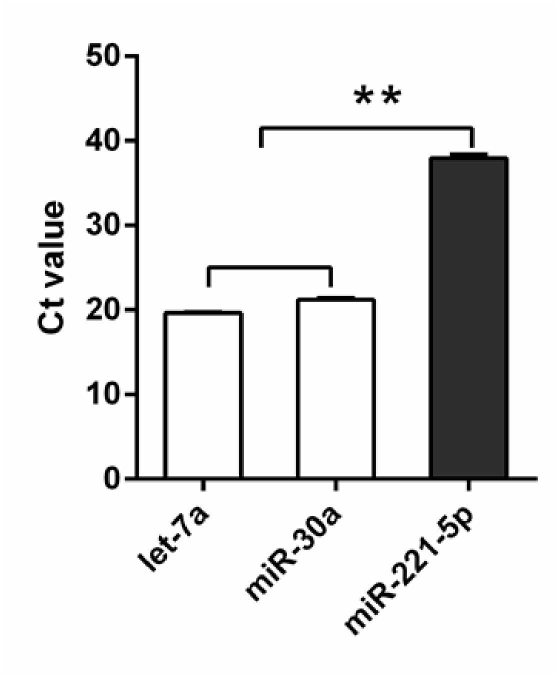

Figure S1. qRT-PCR analysis of let-7a, miR-30a, and miR-221-5p expression levels in PM-sEVs.

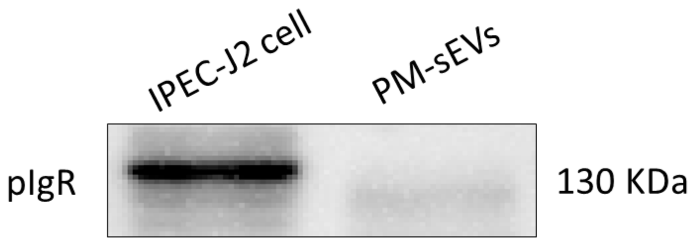

Figure S2. Western blotting analysis of pIgR protein level in IPEC-J2 cell and PM-sEVs.
